# Tackling COVID-19: Can the African continent play the long game?

**DOI:** 10.7189/jogh.10.010339

**Published:** 2020-06

**Authors:** Akaninyene Otu, Bassey Ebenso, Ronald Labonte, Sanni Yaya

**Affiliations:** 1 Department of Infection and Travel Medicine, Leeds Teaching Hospitals NHS Trust, Leeds, UK; 2 Nuffield Centre for International Health and Development, Leeds Institute of Health Sciences, University of Leeds, Leeds, UK; 3 School of Epidemiology and Public Health, University of Ottawa, Ottawa, Ontario, Canada; 4 School of International Development and Global Studies, University of Ottawa, Ottawa, Ontario, Canada; 5 The George Institute for Global Health, The University of Oxford, Oxford, UK

Events have progressed with dizzying rapidity since the World Health Organization (WHO) was first alerted to cases of severe pneumonia in the Wuhan City of China on December 31^st^ 2019. The novel SARS-CoV-2 coronavirus disease (COVID-19) was declared a pandemic on March 11^th^ 2020. As of April 7^th^, a total of 1.38 million cases of COVID-19 had been diagnosed globally with over 78 000 deaths attributable to the disease [1]. Comparisons have been drawn between COVID-19 and other deadly pandemics such as the 1918 Spanish flu that infected about one-third of the world’s population, killed 40-50 million people and changed the course of history [2]. While it is premature to judge the final death toll of COVID-19, the global response to the pandemic will determine how bad it becomes.

## LATE IN THE GAME OR JUST LATE IN BEING COUNTED?

During the initial stages of the pandemic, it appeared Africa would be spared the burden of COVID-19. However, by April 7^th^, a total of 45 countries within the WHO African region had reported over 7000 cases (although some place it at over 10 000), with at least 292 deaths and 612 people recovered [3]. It remains unclear why there are very few cases of COVID-19 in Africa compared to the numbers in European countries and in Asia. Theories proposed to explain the unexpected low COVID-19 numbers in Africa include the dearth of large-scale testing and reporting, weak travel connections, effective border screening and the (more controversially stated) vulnerability of the virus to Africa’s hot climate. The numbers will undoubtedly rise given reports of community transmission in warmer and humid conditions in Southeast Asia and South America that show evidence of daily rapid increases in new COVID-19 cases.

## CAN AFRICAN HEALTH SYSTEMS RESPOND?

African health systems have often grappled with the challenges posed by disease outbreaks. Over the last decade, 41 African countries (87% of the continent) have had at least one epidemic [4]. Examples include the Ebola epidemic of 2014-16 in West Africa [5], recent Ebola epidemic of 2018-2020 in the Democratic Republic of Congo; and recurrent Lassa fever epidemics in Nigeria [6,7]. Experiences from these outbreaks have led to significant improvements in surveillance, preparedness planning, and clinical and laboratory capacity across the African continent [8,9]. But the challenges inherent in not only diagnosing (testing) or treating serious COVID-19 (due to shortages in hospital beds and medical supplies) extends to strategies aimed at prevention, or “flattening” the curve of new infections.

There are wider benefits of established infection prevention strategies such as handwashing (frequent and proper, meaning the 20-second lathered scrub) and good hygiene practices (sanitizing for infection control) in minimizing the spread of other diseases. Yet, strict handwashing as promoted in China, Europe, and America require remarkable inventiveness to ensure successful implementation in situations where 47% of the 783 million people in Sub-Saharan Africa lack access to clean water [10]. Similarly, self-isolation and other physical distancing practices now widely invoked in high-income countries can be challenging in densely populated urban cities or rural communities typified by over-crowded, poorly ventilated living spaces often occupied by three generations of the same family. Work is urgently needed to bring together (virtually to the extent possible, physically distanced if not) representatives from Ministries of Water Resources, Town Planning Departments, and local communities to design context-informed approaches to hand-washing and distancing. No one-size-fits-all strategy will work, and modelling such behaviour in different contexts, especially crowded townships or more distant rural villages, will tax public health systems to the fullest. Countries with well-developed primary health care networks and community health worker cadres may be in a better position to take on such tasks than those lacking in critical health worker capacities at any or all levels of training or deployment.

## RAPID COLLABORATION NEEDED

There is also an urgent need for broad-based regional collaborations to bolster Africa’s readiness to manage disease outbreaks. The key players can include the Africa Centre for Disease Control, the African Union Commission, the Africa Taskforce for Coronavirus Preparedness and Response (AFTCOR), national public health institutes in Member States, private sector and local businesses. In many high-income countries, all non-essential businesses have been closed for certain periods of time (2 weeks to 2 months or longer, depending on severity of the COVID-19 outbreak and spread). In these countries, queues have been introduced to limit people in food stores to ensure physician distancing, with distancing between people as they wait their turns. Such strategies may be difficult in the many open food markets that characterise Africa, but not wholly impossible. Social education and strategic measures such as increasing the distance between stalls in addition to limiting the number of persons accessing the markets at any given time might work in such contexts. It will be challenging to achieve physical distancing on Africa’s overcrowded public transport systems and traffic jams; walking is commonplace across much of the continent, but will need stronger encouragement with reasonable distancing – admittedly a difficult ask. A combination of intersectoral government/community planning and collaboration and the enactment of some travel restrictions may be viable strategies to address this issue. India’s sudden three-week lockdown and transportation ban, although considered by some a necessary if ill-planned approach in a country lacking the health capacity required to manage COVID-19, is fuelling social discontent and leading to tens of thousands of informal urban workers having to walk hundreds of kilometres back to their villages [11].

## LOCAL INPUTS ESSENTIAL

Such temporary lockdowns, either voluntary or mandatory self-isolation for symptomatic persons or those returning from international travel, have been used as effective strategies to limit the spread of COVID-19 in many high-income countries (HIC). However, slums and informal settlements are common in many African cities as is shared accommodation. Communal water and sanitation facilities dot the African landscape. These physical infrastructures will severely limit people’s abilities to self-isolate effectively thus posing a conundrum for which there are no quick fixes. The choice of working from home is more suited for people who work in offices. For the millions of Africans engaged in trading and businesses that are not limited to offices, working from home is just not an option. In the absence of robust economic packages to support the populace (even India is attempting to model what many high-income countries are undertaking) the stay-at-home directives in Africa are very likely to fail and alternative solutions are required. The reality is that in many African countries, physical distancing remains a privilege only a minority can afford [12].

The double burden of infectious and chronic diseases which constitutes major causes of morbidity and mortality in Sub-Saharan Africa [13] have stimulated the emergence of new players, processes and institutions aiming at mitigating their effect. Global health diplomacy, perhaps incentivized by these new players and processes, may be a powerful tool in addressing global health crises such as pandemics [14]. Cuba, for example, continues its long practice of medical diplomacy through increased deployment of its doctors in scores of countries, most recently COVID-19 ravaged Italy [15]. Other countries, including isolated First Nations communities in Canada, are soliciting similar Cuban health diplomacy. China has used health diplomacy for some years in strengthening its economic ties with Africa [16], and is now sending supplies to Africa (and other countries) following a successful decline in its own COVID-19 cases [17]. Health remains neglected in long-term foreign policy priorities of many African countries, which can limit its own intra-continental capacities for rapid mutual assistance. The COVID-19 pandemic, however, might provide a unique opportunity to strengthen the continent’s regional health security measures (surveillance, control, treatment/mitigation, regulation).

## AN EPIDEMIC OF WEAK PUBLIC FINANCING

There are a few signs of the increasing resilience of African health systems to manage outbreaks [18]. However most African countries are still spending below their former Abuja agreement (15% of public revenue on health) [19] with parallel emphasis on the need to prioritize primary health care (PHC) expenditures. Overall tax rates as a percentage of gross domestic product (GDP) remain low in many African countries (averaging 23% in sub-Saharan Africa) compared to high-income countries (53% in North America) [20]. Although some African countries have begun to record positive improvements in tax rates (albeit much of it via regressive consumption taxes), the GDP of many countries is generally too low for the tax levy to make a huge difference domestic financing of a viable health system, at least in any short-term. Most of the continent continues to rely on external funding, which is likely to recede as donor countries incur massive neo-Keynesian style rescues of their own economies. African countries have also not done enough to stem capital flight. Developing countries have experienced investment (capital) outflows since the start of the COVID-19 crisis far exceeding losses following the 2008 global financial crisis [21]. The longer-term implications of the continued reliance on foreign investment, loans, and supply chains to meet basic human/health needs and economic security need to be carefully considered, given the increase in sub-Saharan African external debt from USD236 billion to USD635 billion in 2019 [21]. Global financial interdependencies will exert powerful negative effects on Africa’s capacity to address economic impacts of health emergencies.

## COLLAPSING GLOBAL SUPPLY CHAINS

**Figure:**
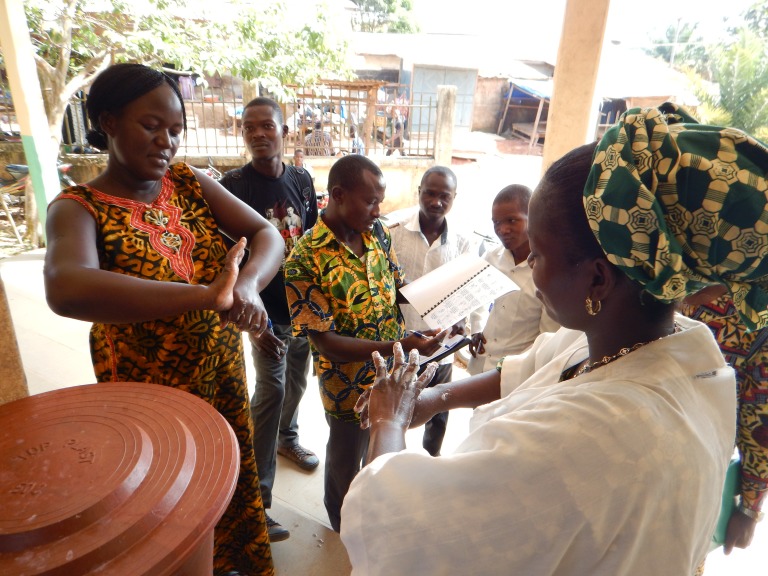
Photo: Training proper hand-washing techniques (CDC/ Conne Ward-Cameron).

Experts already predict that the economic fallout of COVID-19 for the continent will be severe, given that the estimated economic and social cost of the 2014 Ebola crisis in West Africa was US$53 billion [22]. The rapid spread of the COVID-19 across the world, combined with China’s key role in the global economy (representing 18% of global GDP) and its large share of African infrastructure and capital projects (the lifeline of many African countries), will make it difficult for countries to sustain their own economic role in global manufacturing and supply chains.

The world’s higher-income countries are deploying a range of prescriptive economic tools and policy initiatives that have been dubbed “COVID-Keynesianism”. This term describes increased government expenditure and lower interest rates in a bid to compensate for a reduction in economic demand. Since January 2020, interest rates have been cut by more than 0.5 percentage points by many countries and central banks have introduced innovative schemes to free up money to purchase bonds [23]. Some countries have made credit guarantees worth as much as 15% of GDP. The value of these interventions is in the trillions of dollars. The US rescue package alone is worth as much as the entire economic output of Africa in 2019 [24]. The intent is to stimulate to consumption in order to ensure continued production, although much of this latter will likely be focused inwards.

## AFRICA’S RESPONSE TRIES TO FOLLOW OTHER COUNTRIES’ LEADS BUT…

Africa’s response to the economic realities of the contagion is slowly starting to be defined, following many of the steps taken by wealthier nations further along the pandemic curve. Most African countries have instituted some form of temporary lockdown, including closures of schools and non-essential businesses, travel restrictions, bans on gatherings of more than a few people, promotion of physical distancing, and stay-home advisories. Sometimes these measures are being enforced in violent ways which may ironically incentivize people to flee to rural areas, potentially infecting others. Importantly, human rights, communitarianism, and civility should not be sacrificed to excessive restraints too easily used by those with dictatorial ambitions, regardless of which country they may claim to lead. Some African countries have also adopted tax, stimulus, and financial support measures similar to nations in Europe, Asia, and the Americas, albeit not on the same scale. Whether the continent can play the long game of managing the combined public health and socioeconomic impacts of COVID-19 remains to be seen.

Even as the rich world copes with its own upheavals, humanitarian, ethical, and health-self-interest (global health security) demands that it does not neglect the financial and health needs of a continent that has supplied it with much of its wealth over past years and centuries. At a minimum, and as African finance ministers called for on March 23rd, interest payments on the continent’s high foreign debt burden should be suspended, including principal payments for countries facing the greatest fiscal challenges [25]. Intergovernmental funding efforts are also needed, such as the WHO’s call for a COVID-19 Response Fund with pledges now nearing the target of US$675 million in a unique crowd-funding effort in which the WHO is pitching for individual as well as governmental donations [26]. How much new donor support is needed is moot; much depends on how deeply and for how long the pandemic runs. By one estimate, the sub-Saharan Africa region alone will need US$100 billion if it is to come even close to the average level of fiscal stimulus the rich world (the G20) is giving its own citizens [27]. This figure may appear large but it is less than the 2% of what the G20 countries have already committed to themselves.

## REMEMBER THE CRISES BEHIND THE CRISIS

Finally, beneath the immediacy of the pandemic and its likely sweep across Africa lie four decades of a rapacious model of globalisation which accelerated the climate change catastrophe and led to unprecedented levels of wealth and power inequalities [28]. In Africa’s rush to stave off the SARS-CoV-2 outbreak, it would be unwise to forget the existential crises that defined the ‘normal’ world to which we now seem keen to return.
